# Lassa Virus in Multimammate Rats, Côte d’Ivoire, 2013

**DOI:** 10.3201/eid2108.150312

**Published:** 2015-08

**Authors:** Leonce Kouadio, Kathrin Nowak, Chantal Akoua-Koffi, Sabrina Weiss, Bernard K. Allali, Peter T. Witkowski, Detlev H. Krüger, Emmanuel Couacy-Hymann, Sébastien Calvignac-Spencer, Fabian H. Leendertz

**Affiliations:** Robert Koch Institut, Berlin, Germany (L. Kouadio, K. Nowak, S. Weiss, S. Calvignac-Spencer, F.H. Leendertz);; Laboratoire Central de la Pathologie Animal, Bingerville, Côte d’Ivoire (L. Kouadio, E. Couacy-Hymann);; Université Alassane Ouattara de Bouake, Bouake, Côte d’Ivoire (C. Akoua-Koffi);; European Centre for Disease Prevention and Control, Stockholm, Sweden (S. Weiss);; Public Health England, London, UK (S. Weiss);; Institut Pasteur de Côte d'Ivoire (B.K. Allali);; Charité School of Medicine, Berlin (P.T. Witkowski, D.H. Krüger)

**Keywords:** Lassa fever, Lassa virus, viruses, zoonoses, multimammate rat, Côte d’Ivoire, Africa

Lassa fever is a zoonosis caused by Lassa virus (LASV; family Arenaviridae, genus Lassavirus). The primary reservoir of LASV is the multimammate rat (Mastomys natalensis), which is found throughout sub-Saharan Africa. LASV outbreaks among humans occur only in West Africa in 2 noncontiguous areas: 1 in Guinea, Liberia, and Sierra Leone; and 1 in Nigeria. Rare cases and evidence of exposure of humans have been documented in neighboring countries (i.e., Benin, Burkina Faso, Côte d’Ivoire, Ghana, Mali, and Togo) (*1*). LASV RNA has been detected in only 4 patients: 1 in Germany who had traveled in Burkina Faso, Côte d’Ivoire, and Ghana (*2*); 1 in the United Kingdom who had returned from Mali (*3*); and 2 in Ghana, for whom no viral sequence was available because detection was performed by reverse transcription PCR only (*4*). In the region in Mali where the patient from the United Kingdom was infected, identical LASV sequences were found in multimammate rats (*5*). The sequence of the strain identified from the patient in Germany, who was designated AV, is the closest known relative of the clade formed by sequences from Mali (*5*). However, LASV was not found in its natural host in any of the countries visited by patient AV (*6**,**7*).

For a study investigating zoonotic pathogens in rural habitats, we caught small mammals in 3 ecologic zones of Côte d’Ivoire: 1) dry bushland in northern Côte d’Ivoire, around Korhogo (*2*); semiarid bushland in central Côte d’Ivoire, around Bouake; and rainforest in southwestern Côte d’Ivoire, near the Taï National Park (*3*) (Technical Appendix Figure). Traps were installed within and around 15 villages and enabled the capture of 27 eulipotyphlans and 254 rodents during August–October 2013. Animals were assigned at the genus level in the field on the basis of morphology. For 88% of them, assignment could later be refined to the species level by sequencing a fragment of the mitochondrial cytochrome *b* gene. A total of 14 animal species representing 8 genera were detected. All host sequences were deposited in Dryad (http://www.datadryad.org/; Technical Appendix Table 1). Multimammate rats were the dominant commensals at all sampling locations, comprising 64.5% of the overall sample (Technical Appendix Figure).

Tissue samples were collected from all animals according to standard protocols. Total nucleic acids were extracted from lung samples and tested for the presence of LASV RNA by using a real-time PCR system amplifying a 400-bp fragment of the large genomic segment (*8*) (Technical Appendix). LASV RNA was detected in 4 of 18 specimens of *M. natalensis* captured in Gbalôhô, near Korhogo (online Technical Appendix Figure). This site is much farther north in Côte d’Ivoire than previously examined sites (*6*). The 4 PCR-positive animals were 3 males and 1 female that were all captured indoors, 3 in the same house. PCR products were sequenced according to the Sanger method (GenBank accession nos. LN823982–LN823985). According to phylogenetic analyses performed in maximum likelihood and Bayesian frameworks (Technical Appendix), LASV sequences identified in multimammate rats from Côte d’Ivoire formed a robust clade with sequences from the human AV strain and the LASV infecting multimammate rats in southern Mali (bootstrap 97, posterior probability 1.00; Figure). This phylogenetic placement opens up the possibility that patient AV was infected during her travel through Côte d’Ivoire, possibly in or near the city of Korhogo. Tip date calibration of Bayesian analyses showed that the most recent common ancestor of all LASV sequences from Côte d’Ivoire and Mali circulated ≈90 years ago (Figure; Technical Appendix Table 2).

**Figure:**
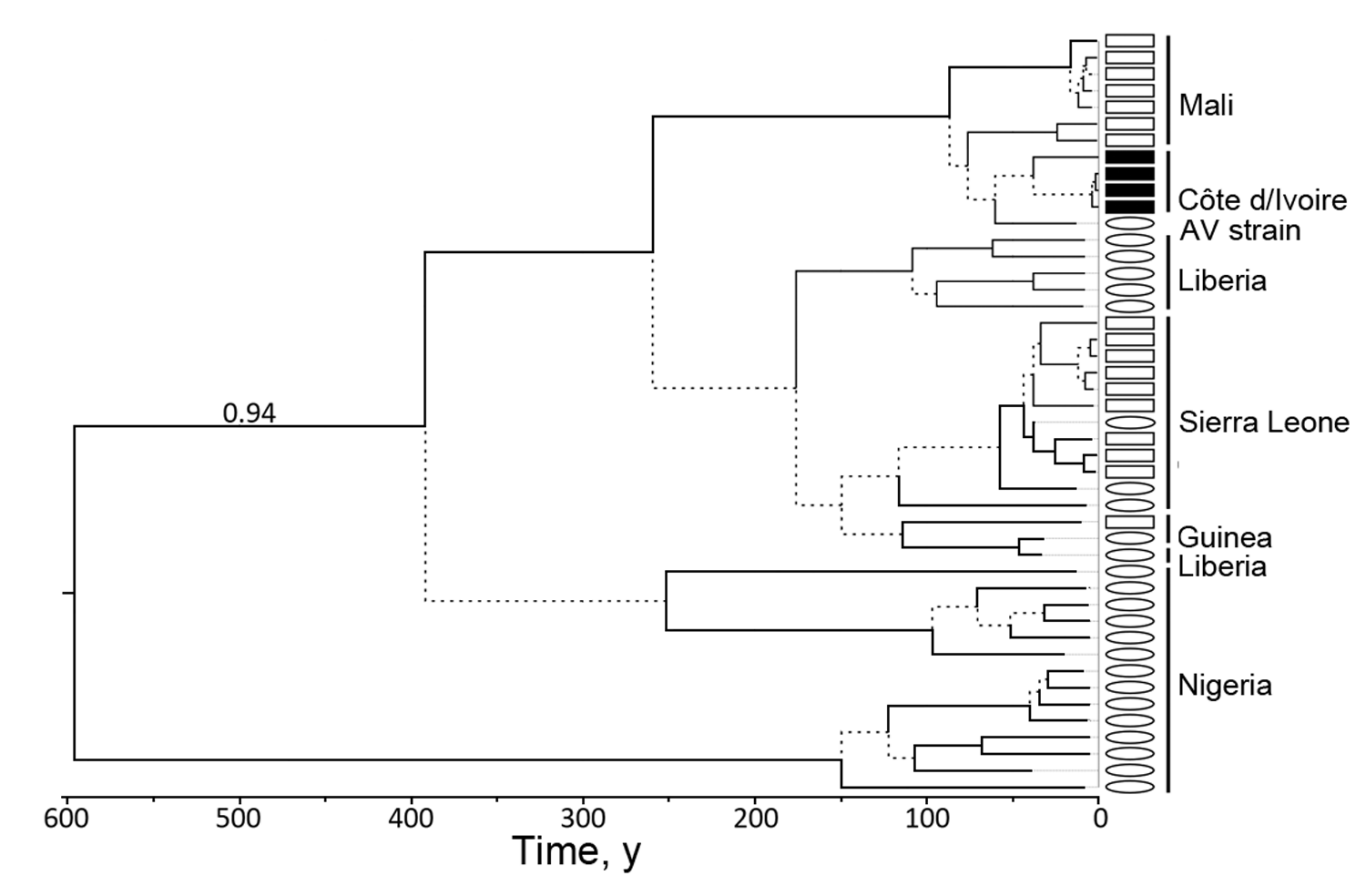
Bayesian chronogram of Lassa virus (LASV) sequences determined on the basis of a fragment of the large genomic segment. Branches receiving posterior probability values <0.95 and bootstrap values <50 (poorly supported) are dashed. LASV sequences of human origin are indicated by ovals, and those of multimammate rats are indicated by squares. Sequences reported in this study are indicated by black squares. This tree was built under the assumption of a molecular clock and is therefore rooted. The numerical value on the tree’s most basal branch is the root posterior probability of this branch; it supports the notion that LASV sequences from Nigeria and other countries are not reciprocally monophyletic. GenBank accession nos. of sequences used for phylogenetic analyses are shown in Technical Appendix Table 2. AV strain indicates the strain from a German patient.

Further studies will be needed to investigate the geographic distribution of LASV in Côte d’Ivoire and the frequency of human infections. The current lack of diagnosed cases in the area may be caused by underreporting. Sensitization campaigns are needed to increase awareness of the risk for LASV infection among the local population and to improve detection of cases by health workers.

Technical Appendix Detailed methods and results, Lassa virus in multimammate rats, Côte d’Ivoire, 2013.
