# Supplementary material for: Lassa Virus in Multimammate Rats, Côte d’Ivoire, 2013
Source: Emerg Infect Dis. 2015 Aug;21(8):1481–3. doi: 10.3201/eid2108.150312 (PMC4517737; doi:10.3201/eid2108.150312)
Supplement: Technical Appendix — Detailed methods and results, Lassa virus in multimammate rats, Côte d’Ivoire, 2013. [file 15-0312-Techapp-s1.pdf]

Article DOI: http://dx.doi.org/10.3201/eid2108.150312

Lassa Virus in Multimammate Rats, Côte d'Ivoire, 2013

Technical Appendix

Experimental Procedures and Results

Sampling

Small mammals were captured during August–October 2013 in 15 villages in northern, central, and southwestern Côte d'Ivoire (Technical Appendix Figure). Animals were trapped with Sherman traps (Pet Factory, Hülsede, Germany). Following methods of Fichet-Calvet et al. (*1*), we placed 50 traps inside houses (2 trap set in each room) and 40 traps in the cultivations and forests surrounding every village for 3 consecutive nights (*1*). Traps were baited daily with a mixture of peanuts, dry fish, and wheat flower.

All animals were anesthetized and euthanized with ether, according to animal welfare guidelines. A predefined set of morphologic features was recorded for each animal (sex, age category, weight, length of head and body, length of tail, and length of hind foot). At this stage, a preliminary assignment to the genus level was performed. Whole blood and tissue samples were taken in the following order: liver, spleen, lung, kidney, and intestine; intestine was sampled last to minimize risk of fecal contamination of the other organs. All samples were immediately preserved in liquid nitrogen, transported on dry ice, and stored at –80°C upon arrival at the Robert Koch Institute (Berlin, Germany).Handling and necropsies of the animals were performed only by trained workers who applied appropriate safety measures.

Molecular Biology

Total nucleic acids were extracted from lung samples by using a NucleoSpin kit (Macherey-Nagel, Düren, Germany). Reverse-transcription PCR was performed by using the SuperScript II Reverse transcription kit (Invitrogen, Carlsbad, CA, USA) and random hexamer primers (Bioline, London, UK).

Five microliter complementary DNA (cDNA) samples were tested for the presence of Lassa virus genetic material by using a PCR assay targeting a ca. 400 bp fragment of the RNA polymerase (L) gene (*2*). PCR reactions (total volume 25 µL) were prepared to contain 4 U Platinum *Taq* (Invitrogen), 1X PCR buffer, 1.5 mM $MgCl_2$, 200 µM dNTPs, 0.3 µM forward primers (LVL3359A, LVL3359D and LVL3359G), and 0.6 µM reverse primers (LVL3754A and LVL3754D). Cycling conditions were as follows: 95°C for 2 minutes; 45 cycles at 95°C for 20 seconds, 55°C for 45 seconds, and 72°C for 60 seconds; and final extension at 72°C for 10 minutes.

One microliter nucleic acid extract was also used to amplify a ca. 800 bp fragment of the mitochondrial cytochrome *b* gene of the host (*3*). PCR reactions (total volume 25µL) were prepared to contain 2 U Platinum *Taq* (Invitrogen), 1X PCR buffer, 4 mM $MgCl_{2,}$ 200 µM dNTPs, and 0.2 µM of each primer (L14724 and H15506). Cycling conditions were as follows: 95°C for 5 minutes; 40 cycles at 95°C for 30 seconds, 52°C for 30 seconds, and 72°C for 45 seconds; and final extension at 72°C for 10 minutes.

All PCR products were sequenced on both strands according to Sanger's method. Sequence identity was confirmed with BLAST (National Center for Biotechnology Information, Bethesda, MD, USA) (*4*). BLAST results were further used to assign host sequences to species in combination with genus-level morphologic identification and occurrence data derived from the International Union for Conservation of Nature Red List database (http://www.iucnredlist.org/; Technical Appendix Table 1).

Isolation of Virus from Positive Samples Not Attempted Thus Far

Lassa Virus Phylogenetic Analyses

A dataset of the sequences generated in this study, selected reference sequences derived from infected humans, and all sequences derived from multimammate rats were reduced to unique haplotypes by using ALTER (*5*). The haplotyped dataset comprised 53 sequences, including 46 with known dates of collection. Further analyses were performed on the full haplotyped dataset (D_{53}), and the same dataset reduced to sequences with known dates of collection (D_{46}). Technical Appendix Table 2 describes the sequences comprised in D_{53} (GenBank accession numbers, host, and place and date of sample collection).We used

jModelTest v2.1.4 (*6*) and the Bayesian information criterion to select the model of nucleotide substitution that best fit the datasets (HKY+G). Maximum likelihood trees were inferred by using PhyML v3.0 (*7*), as implemented on the PhyML webserver (*8*). Tree search started from a BioNJ tree and used the BEST algorithm; branch robustness was assessed by nonparametric bootstrapping (250 pseudo-replicates). Bayesian analyses were performed by using BEAST v1.8.0 (*9*). For both datasets, analyses were performed under an uncorrelated relaxed molecular clock (lognormal) and 2 different demographic models (constant population size and SkyGrid). For any analysis, 2 Bayesian Markov chain Monte Carlo analyses were run; convergence was checked visually within and between individual runs by using Tracer v1.6 (http://tree.bio.ed.ac.uk/software/tracer/), and appropriate sampling of the posterior was documented (effective sample sizes >200 for all model parameters).

The relaxed clocks were calibrated as follows: for D_{53}, a strong prior was placed on the root age (normal distribution of real mean 750 years, standard deviation 130 years; 95% highest-posterior density: 500–100 years) on the basis of a published estimate of Lassa virus ancestry (*10*); for D_{46}, tip dates were used to inform the evolutionary rate. Both calibrations ended with very similar substitution rate estimates (Table 3). Control experiments sampling only from the prior showed that divergence dates were informed by sequences; however, these results should be considered with care as no real clock-likeness signal could be detected from the maximum likelihood tree when it was analyzed with Path-O-Gen v1.4 (http://tree.bio.ed.ac.uk/software/pathogen/).

The output of the Bayesian Markov chain Monte Carlo runs was combined by using LogCombiner v1.8.0 (distributed with BEAST). The resulting tree file was analyzed with RootAnnotator (*11*), which itself used TreeAnnotator v1.8.0 (distributed with BEAST); this analysis enabled plotting of root posterior probabilities along with other values of interest. The final tree was plotted by using FigTree v1.4 (http://tree.bio.ed.ac.uk/software/figtree/) and further edited with Inkscape (https://inkscape.org/en/).

References

<jrn>1. Fichet-Calvet E, Lecompte E, Koivogui L, Soropogui B, Dore A, Kourouma F, et al. Fluctuation of abundance and Lassa virus prevalence in *Mastomys natalensis* in Guinea, West Africa. Vector Borne Zoonotic Dis. 2007;7:119–28. PubMed http://dx.doi.org/10.1089/vbz.2006.0520</jrn>

<jrn>2. Lecompte E, Fichet-Calvet E, Daffis S, Koulemou K, Sylla O, Kourouma F, et al. *Mastomys natalensis* and Lassa fever, West Africa. Emerg Infect Dis. 2006;12:1971–4. PubMed http://dx.doi.org/10.3201/eid1212.060812</jrn>

<jrn>3. O'Brien J, Mariani C, Olson L, Russell AL, Say L, Yoder AD, et al. Multiple colonisations of the western Indian Ocean by *Pteropus* fruit bats (Megachiroptera: Pteropodidae): the furthest islands were colonised first. Mol Phylogenet Evol. 2009;51:294–303. PubMed http://dx.doi.org/10.1016/j.ympev.2009.02.010</jrn>

<jrn>4. Altschul SF, Gish W, Miller W, Myers EW, Lipman DJ. Basic local alignment search tool. J Mol Biol. 1990;215:403–10. PubMed http://dx.doi.org/10.1016/S0022-2836(05)80360-2</jrn>

<jrn>5. Glez-Pena D, Gomez-Blanco D, Reboiro-Jato M, Fdez-Riverola F, Posada D. ALTER: program-oriented conversion of DNA and protein alignments. Nucleic Acids Res. 2010;38:W14–8.</jrn>

<jrn>6. Darriba D, Taboada GL, Doallo R, Posada D. jModelTest 2: more models, new heuristics and parallel computing. Nat Methods. 2012;9:772. PubMed http://dx.doi.org/10.1038/nmeth.2109</jrn>

<jrn>7. Guindon S, Dufayard JF, Lefort V, Anisimova M, Hordijk W, Gascuel O. New algorithms and methods to estimate maximum-likelihood phylogenies: assessing the performance of PhyML 3.0. Syst Biol. 2010;59:307–21. PubMed http://dx.doi.org/10.1093/sysbio/syq010</jrn>

<jrn>8. Guindon S, Lethiec F, Duroux P, Gascuel O. PHYML Online–a web server for fast maximum likelihood-based phylogenetic inference. Nucleic Acids Res. 2005;33:W557–9. PubMed http://dx.doi.org/10.1093/nar/gki352</jrn>

<jrn>9. Drummond AJ, Suchard MA, Xie D, Rambaut A. Bayesian phylogenetics with BEAUti and the BEAST 1.7. Mol Biol Evol. 2012;29:1969–73. PubMed http://dx.doi.org/10.1093/molbev/mss075</jrn>

<jrn>10. Lalis A, Leblois R, Lecompte E, Denys C, Ter Meulen J, Wirth T. The impact of human conflict on the genetics of *Mastomys natalensis* and Lassa virus in West Africa. PLoS ONE. 2012;7:e37068. PubMed http://dx.doi.org/10.1371/journal.pone.0037068</jrn>

<jrn>11. Calvignac-Spencer S, Schulze JM, Zickmann F, Renard BY. Clock rooting further demonstrates that Guinea 2014 EBOV is a member of the Zaire lineage. PLoS Curr. 2014;6:pii. PubMed</jrn>

Technical Appendix Table 1. Small mammal species determination of the 248 animals that could be assigned to the species level (i.e., 88% of the sampling)

Order	Species	Bouaké	Korhogo	Täi	Total (%)
Eulipotyphla	*Crocidura buettikoferi*			2	2 (0.8)
	Crocidura jouvenetae	1			1 (0.4)
	Crocidura olivieri	9	4	1	14 (5.6)
	Crocidura theresae	1			1 (0.4)
Rodentia	*Lophuromys sikapusi*		2		2 (0.8)
	Mastomys natalensis	54	65	41	160 (64.5)
	Mus mattheyi	1			1 (0.4)
	Mus minutoides	9	1	16	26 (10.5)
	Mus setulosus	9		1	10 (4.0)
	Praomys daltoni	1			1 (0.4)
	Praomys rostratus		4	6	10 (4.0)
	Rattus rattus	1		13	14 (5.6)
	Taterillus gracilis		2		2 (0.8)
	Uranomys ruddi	4			4 (1.6)
Total (%)		90 (36.3)	78 (31.5)	80 (32.3)	

Technical Appendix Table 2. Sequences used for phylogenetic analyses of Lassa fever viruses in multimammate rats, Côte d'Ivoire, 2013

GenBank accession no.	Host	Place of collection	Date of collection*
AY179171	*Homo sapiens*	Germany	2000
AY628200	*Homo sapiens*	Guinea	1981
AY628204	*Homo sapiens*	Liberia	?
AY693638	*Homo sapiens*	Liberia	1980
AY870334	*Homo sapiens*	Liberia	2004
GU979509	*Homo sapiens*	Liberia	2005
GU979510	*Homo sapiens*	Liberia	2005
GU979511	*Homo sapiens*	Liberia	2005
GU979512	*Homo sapiens*	Liberia	2005
AY179175S1	*Homo sapiens*	Nigeria	2000
AY693637	*Homo sapiens*	Nigeria	2004
AY693639	*Homo sapiens*	Nigeria	1993
AY693640	*Homo sapiens*	Nigeria	1974
GU481057	*Homo sapiens*	Nigeria	2005
GU481062	*Homo sapiens*	Nigeria	2007
GU481065	*Homo sapiens*	Nigeria	2008
GU481067	*Homo sapiens*	Nigeria	2008
GU481069	*Homo sapiens*	Nigeria	2008
GU481071	*Homo sapiens*	Nigeria	2008
GU481073	*Homo sapiens*	Nigeria	2008
GU481075	*Homo sapiens*	Nigeria	2008
GU481077	*Homo sapiens*	Nigeria	2008
GU481079	*Homo sapiens*	Nigeria	2008
AY179172	*Homo sapiens*	Sierra Leone	2000
AY363905	*Homo sapiens*	Sierra Leone	?
AY363906	*Homo sapiens*	Sierra Leone	?
AY363907	*Homo sapiens*	Sierra Leone	?
AY363908	*Homo sapiens*	Sierra Leone	?
AY363909	*Homo sapiens*	Sierra Leone	?
AY363910	*Homo sapiens*	Sierra Leone	?
GU979507	*Homo sapiens*	Sierra Leone	2006
U63094	*Homo sapiens*	Sierra Leone	1976
LN823982	*Mastomys natalensis*	Cote d'Ivoire	2013
LN823983	*Mastomys natalensis*	Cote d'Ivoire	2013
LN823984	*Mastomys natalensis*	Cote d'Ivoire	2013
LN823985	*Mastomys natalensis*	Cote d'Ivoire	2013
GU979513	*Mastomys natalensis*	Guinea	2003
GU573541	*Mastomys natalensis*	Mali	2009
GU573545	*Mastomys natalensis*	Mali	2009
GU573546	*Mastomys natalensis*	Mali	2009
KF478760	*Mastomys natalensis*	Mali	2012
KF478761	*Mastomys natalensis*	Mali	2012
KF478763	*Mastomys natalensis*	Mali	2012
KF478764	*Mastomys natalensis*	Mali	2012
KM822110	*Mastomys natalensis*	Sierra Leone	2010

GenBank accession no.	Host	Place of collection	Date of collection*
KM822112	*Mastomys natalensis*	Sierra Leone	2010
KM822114	*Mastomys natalensis*	Sierra Leone	2009
KM822116	*Mastomys natalensis*	Sierra Leone	2012
KM822119	*Mastomys natalensis*	Sierra Leone	2012
KM822121	*Mastomys natalensis*	Sierra Leone	2012
KM822123	*Mastomys natalensis*	Sierra Leone	2012
KM822125	*Mastomys natalensis*	Sierra Leone	2012
KM822129	*Mastomys natalensis*	Sierra Leone	2011

*?, no precise date of collection provided in the original publication.

Technical Appendix Table 3. Substitution rate, divergence dates, and root position across models used in a study of Lassa fever viruses in multimammate rats, Côte d'Ivoire, 2013*

		Median rate [95% HPD](subst.site^{-1}.year^{-1})	Divergence date [95% HPD]		Root posterior probability (MCC tree root/Nigeria vs. rest)
Calibration	Demography		Human CI + rat CI	Mali CI	
Root	Constant size	7.6^{-4} [3.9^{-4}-1.3^{-3}]	56 [18–104]	89 [32–162]	0.887/0.074
	Skygrid	6.7^{-4} [3.3^{-4}-1.2^{-3}]	64 [25–122]	102 [38–185]	0.890/0.074
Tips	Constant size	8.1^{-4} [5.1^{-4}-1.1^{-3}]	60 [36–92]	87 [53–132]	0.943/0.041
	Skygrid	7.9^{-4} [4.9^{-4}-1.1^{-3}]	63 [37–98]	90 [54–138]	0.929/0.047

*CI, Côte d'Ivoire; HPD, highest posterior density; MCC, maximum clade credibility.

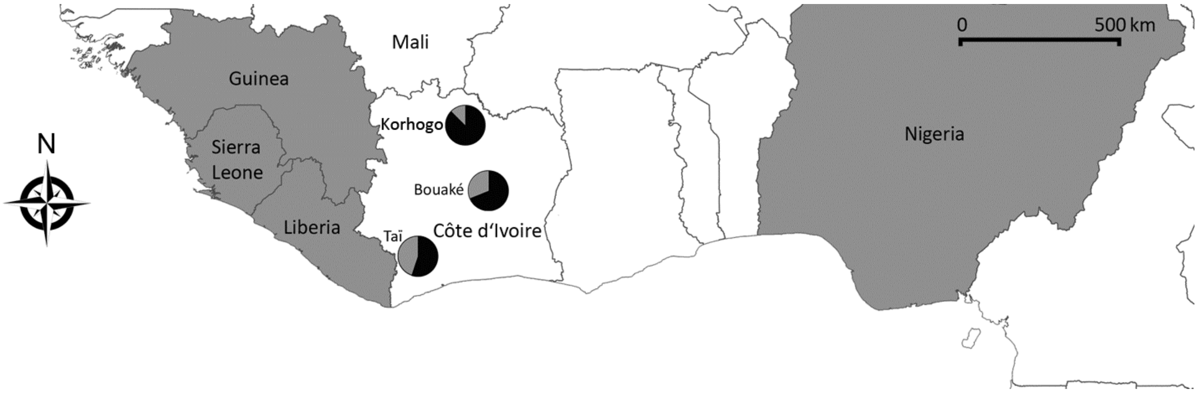

Technical Appendix Figure. Map of West Africa showing regions endemic for Lassa fever (LASV) and sampling sites for study of LASV in multimammate rats, Côte d'Ivoire, 2013. Pie charts in map represent sampling sites in Côte d'Ivoire (Global Positioning System coordinates: 30 P 211825 1045219, Korhogo; 30 N 276206 849368, Bouake; 29 N 670895 649275, Täi). Within the charts, samples from *Mastomys* sp. rats are indicated in black; those from other rodents are in gray. On the map, countries in which LASV is endemic are shaded in gray.
